# Toward robust social media sentiment for SMEs: a comparative study of dictionary-based and machine learning approaches with insights for hybrid methodologies

**DOI:** 10.3389/fdata.2025.1594374

**Published:** 2026-04-01

**Authors:** Heru Susanto, Aida Sari Omar, Alifya Kayla Shafa Susanto, Desi Setiana, Leu Fang-Yie, Junaid M. Shaikh, Asep Insani, Uus Khusni, Rachmat Hidayat, Akbari Indra Basuki, Iwan Setiawan

**Affiliations:** 1 School of Business, Universiti Teknologi Brunei, Bandar Seri Begawan, Brunei; 2 National Research and Innovation Agency (BRIN), Jakarta, Indonesia; 3 Center for Green Technology and Sustainability, Universiti Teknologi Brunei, Bandar Seri Begawan, Brunei; 4 School of Computing and Informatics, Universiti Teknologi Brunei, Bandar Seri Begawan, Brunei; 5 University of Applied Sciences and Arts Northwestern Switzerland, Zurich, Switzerland; 6 Digital Psychology, Universiti Brunei Darussalam, Bandar Seri Begawan, Brunei; 7 Department of Information Management, Tunghai University, Taichung City, Taiwan; 8 University of Technology Brunei, Bandar Seri Begawan, Brunei

**Keywords:** sentiment analysis, social media, SMEs, dictionary-based methods, machine learning, hybrid framework

## Abstract

Small and Medium-sized Enterprises (SMEs) increasingly rely on social media to engage customers, promote products, and enhance workplace collaboration. Customer opinions expressed through comments and posts on platforms such as Facebook and Instagram represent valuable insights, yet their informal and context-specific nature—often characterized by slang, misspellings, and bilingual usage—poses challenges for automated sentiment analysis. This study addresses this gap by comparatively evaluating dictionary-based and machine learning approaches to sentiment classification for SMEs' social media content. Data were collected from a diverse set of SMEs across multiple industries, with a substantial volume of customer comments extracted and pre-processed through tokenization, normalization, stop-word removal, and stemming. A customized dictionary was developed to account for local language variations, while Naïve Bayes and Support Vector Machine (SVM) models were employed as supervised classifiers. The findings indicate that dictionary-based methods, while simple and interpretable, struggle with accuracy when processing informal and localized language, whereas machine learning approaches deliver higher overall performance but require extensive preprocessing and tuning. Moreover, the study highlights the potential of hybrid frameworks that combine the interpretability of dictionary-based models with the adaptability of machine learning classifiers. This research contributes both practically and theoretically by (i) demonstrating the limitations of applying generic sentiment analysis tools in localized SME contexts, (ii) proposing a hybrid sentiment analysis framework tailored to SMEs, and (iii) offering empirical evidence to support digital transformation strategies for SMEs in resource-constrained environments. Ultimately, accurate sentiment analysis can enable SMEs to refine business strategies, strengthen customer engagement, and achieve sustainable growth in the digital economy.

## 1 Introduction

Small and Medium Enterprises (SMEs) continue to play a vital role in driving GDP growth and employment worldwide. Recent evidence (Gottschalk and Weise, 2023) highlights that SMEs contribute over 90% of all businesses globally and account for 60–70% of total employment. In the current digital economy, SMEs are increasingly leveraging **social media platforms** as strategic tools to enhance brand visibility, foster customer engagement, and promote innovation (Orji et al., 2022; Contreras et al., 2022).

Since the early 2000s, the advent of Web 2.0 technologies has transformed communication and marketing dynamics. According to Chaffey (2022), there were approximately **4.62 billion internet users worldwide** by January 2022, representing a continuous rise from 4.2 billion in 2021. This expansion has enabled SMEs of all scales to actively participate on platforms such as **Facebook, Instagram, TikTok, and X (formerly Twitter)**, directly connecting with tech-savvy consumers. Social media thus provides cost-effective opportunities for **digital marketing, product feedback, and customer relationship management**, especially for small firms with limited resources (Vohra and Teraiya, 2021).

However, the increasing flow of user-generated content poses analytical challenges. Customers continuously post opinions, reviews, and comments about businesses, creating vast unstructured datasets that contain valuable insights. Extracting these insights efficiently requires **sentiment analysis**, a branch of natural language processing (NLP) that classifies public attitudes toward brands, products, or services. Despite its potential, existing sentiment-analysis tools often underperform in low-resource and multilingual contexts such as Brunei, where mixed Malay–English communication, local slang, and informal spelling dominate online discourse (Meske and Stieglitz, 2013; Schwaiger et al., 2017; Polanska, 2016).

This study addresses these limitations by examining and comparing **dictionary-based** and **machine-learning-based** sentiment-analysis methods in the SME context. It proposes a **hybrid framework** that integrates both approaches to improve accuracy and contextual adaptability, particularly for Bruneian SMEs facing data and resource constraints. The remainder of this paper is structured as follows: Section 2 presents a detailed literature review of recent works (2020–2025), Section 3 outlines the research methodology, Section 4 discusses results and model performance, and Section 5 elaborates on theoretical and managerial implications.

### 1.1 Background of study

#### 1.1.1 Social media growth

Social media has evolved into a critical component of SME marketing and customer-engagement strategies. It enables two-way communication, knowledge sharing, and trust-building between businesses and consumers (Meske and Stieglitz, 2013; Orji et al., 2022). With low entry barriers and minimal technological requirements, digital platforms allow SMEs—even in small economies like Brunei—to compete effectively by disseminating information, launching promotions, and gaining valuable market insights (Halim et al., 2025). Beyond a communication tool, social media has transformed into a powerful marketing ecosystem that supports **word-of-mouth (WOM) marketing**, brand advocacy, and influencer engagement, helping businesses shape consumer perceptions and behaviors (Miller and Lammas, 2010; Öztamur and Karakadilar, 2014).

Over the past decade, customers have increasingly turned to social media rather than traditional search engines to obtain information about products, services, and organizations (Hernandez et al., 2015). This shift reflects a broader digital transformation in which social media builds trust between companies and consumers in ways that traditional media could not (Deelmann et al., 2004). Platforms such as Facebook, Instagram, and TikTok now serve as key spaces for product discovery, customer reviews, and online reputation management. For instance, in Brunei, Instagram strongly influences purchasing decisions and product reviews among younger consumers, underscoring the role of social media in shaping SME marketing strategies and consumer trust (Polanska, 2016).

Following the COVID-19 pandemic, digital adoption among SMEs surged globally, with many firms transitioning toward **data-driven decision-making** using social-media analytics (Contreras et al., 2022; Gottschalk and Weise, 2023). However, while SMEs actively embrace digital tools, many still lack the technical capacity to analyze the overwhelming volume of unstructured customer feedback generated online. This gap highlights the urgent need for **automated sentiment-analysis frameworks** tailored to SMEs' operational realities, linguistic diversity, and limited IT infrastructure. Such tools can empower businesses to transform social-media data into actionable insights, improve service quality, and strengthen competitiveness in the evolving digital marketplace.

#### 1.1.2 Sentiment analysis

In its most general form, Sentiment analysis is a concept relating to the procedure of evaluating how different people feel about a particular subject. Pang and Lee (2008) has analyzed a comprehensive specification on the primary methodology underlying sentiment classification and analysis. Opinion mining and sentiment analysis both include several key components, the most important of which are the classification of subjectivity and polarity, the identification of opinion sources, and the summarizing of opinions. Some alternative names for sentiment analysis include sentiment polarity, sentiment classification, and opinion mining. The examination of review texts and the attribution of an emotional value to them is one of the primary objectives of sentiment analysis. The strategies can be grouped into three primary classifications: machine learning approach, dictionary-based approach and some would prefer using the hybrid approach which is a mixture of machine learning and dictionary-based approach to analyze sentiments. *Two* methods are frequently utilized for conducting sentiment analysis across all languages: First, there are methodologies founded on unsupervised learning and dependent on lexicons of feelings and emotions. It is a list of phrases that have been categorized into many subcategories according to whether or not they convey a constructive or destructive spirit. Second, methods for supervised learning that begin with the construction of a model with the assistance of training data and then use this model to generate predictions based on data that has been freshly discovered are referred to as “supervised learning strategies.” When conducting a study of customer sentiment, it is necessary to take into account each and every word of the feedback supplied by the consumer.

According to Kumar and Sebastian (2012) it is a subset of text categorization in which emotional tone rather than topic is used as the defining characteristic. The task at hand is to figure out whether a specific section of text reflects a good or negative attitude in relation to a certain topic. When trying to achieve uniformity and consistency throughout a work, a smart place to begin would be to zero in on words that are frequently employed for the purpose of communicating the feelings in question. This can include things like expressions, views, sentiments, emotions, evaluations, beliefs, and hypotheses. As a direct result of this, several research results can be found in the fields of Web mining, Informational retrieval, Text Mining and Natural Language Processing (NLP). Pang et al. (2002) have shown, However, this is not as straightforward as one might assume it to be due to the fact that feelings are frequently conveyed in a cryptic or ambiguous manner. There is a broad diversity of vocabulary that is used to explain sentiment analysis because it is still a very young field of research. One reason for this is because sentiment analysis is still relatively new (Liu, 2012) and a recent rise in attention in the area of sentiment analysis can be attributed to the fact that and have acknowledged the scientific investigations and potential applications afforded by the analysis of emotional language. Furthermore, free-form survey responses provided in natural language format could be analyzed using sentiment classifications, making the analysis of sentiment helpful in applications for corporate intelligence as well as recommendation systems. This study is difficult to differentiate from topic-based categorization because, in contrast to subjects, which are frequently recognized by phrases alone, emotions can be expressed in a more complex manner, making it more difficult to detect. For example, “Who is willing to buy this product” is a sentence that shows no words contained a single negative word. As a result, it appears that comprehending sentiment necessitates more knowledge than the typical categorization of words. Because of the increasing popularity of social media, companies are being urged to monitor conversation among customers on social medias. This is because social media shapes customer preferences through influencing the attitudes and behaviors of customers about what they are talking about. These opinions can be used to assess a company's social media marketing and brand communication strategies, as well as to determine the awareness of the brand and customers' satisfaction with a product or service. Social media is now more frequently used by businesses looking to connect with their target market due to its convenience. Sentiment analysis and text mining have found social media to be a gold mine owing to the widespread practice of discussing one's feelings about a wide variety of topics, products, and services.

#### 1.1.3 Value of sentiment analysis through content analysis

When linguistic and communicative features are relevant to the investigation, content analysis is the most appropriate research method to use. It consists of a coding system that was developed based on certain research issues that were discovered through a study of the applicable studies. This makes it easy to identify the data units that need to be categorized, documented, compared, and analyzed so that a conclusion can be drawn about the content of the communication. It is a method for examining the communications strategy in and of itself, as opposed to the sender's and recipient's respective understandings of the strategy. The use of sentiment analysis to the understanding of a message, comment, or analysis of any current events may be useful in several circumstances. Sentiment analysis is more vital than ever before because of the sheer volume of data available on social media. The huge interest in political news is also an important factor. Making a profit is also not the only motivation for people to go online in search of information or to voice their opinions. Concerning public security, sentiment analysis can help authorities uncover potentially dangerous information ahead of time. By cutting off cyber terrorists' access to the Internet, for example, they would be deprived of a crucial tool in their fight against us.

The usage of sentiment analysis can be beneficial to small and medium-sized businesses because it enables these businesses to provide their online customers with a higher level of service. The newly found customer preferences, on the other hand, could be utilized by businesses and e-commerce websites to gain a deeper understanding of the products and services they offer. Sentiment analysis of social media posts can shed light on user demands, making this information invaluable for developing personalized advertisements to target customers and introducing new product or service prospects. People are able to stay informed about current events and acquire information from all around the country due to the proliferation of social media. Data analytics can be utilized to glean valuable insights from social media platforms, providing a competitive advantage and facilitating better daily business decisions. In order to be successful, small, and medium-sized enterprises (SMEs) especially in Brunei, the need to understand how customers feel about the products and services they offer. Analyzing client sentiment toward a company's brand and its products or services is a powerful tool. Sentiment analysis makes use of text processing, Natural Language Processing (NLP), and other computerized methods to retrieve, prepare the data, and identify subjective data from social media platforms due to their depth, variety, and quantity in order to better comprehend, display, and anticipate the emotions underlying written content.

To provide a clear roadmap for readers, the remainder of this paper is structured as follows:

**Section 2 – Literature Review** This section synthesizes relevant studies on social media analytics and sentiment analysis, focusing on research published between 2020 and 2025. It reviews the theoretical foundations and empirical findings surrounding **dictionary-based**, **machine-learning**, and **hybrid** sentiment-analysis approaches, particularly in SME and multilingual contexts. The section also identifies methodological gaps—such as limited localization, language diversity, and data-quality issues—that justify the present study's focus on Bruneian SMEs.

**Section 3 – Research Methodology** This section outlines the research design and analytical framework adopted in the study. It explains the **data-collection procedures** from Facebook and Instagram pages of five SMEs in Brunei, followed by detailed **data-preprocessing steps** including tokenization, stop-word removal, normalization, and stemming. The section then describes the **classification methods** employed—manual classification, dictionary-based sentiment analysis, and supervised machine-learning models (Naïve Bayes and SVM)—along with the **evaluation metrics** used (accuracy, precision, recall, and F1-score).

**Section 4 – Key Findings** This section presents an overview of the primary empirical outcomes derived from the comparative analysis of the three sentiment-classification approaches. It summarizes key performance results, highlighting observed linguistic patterns such as bilingual expressions, slang, and sarcasm that influence classification accuracy.

**Section 5 – Data Analysis and Discussion** This section combines the analytical and interpretive components of the study. It provides an in-depth quantitative and qualitative examination of the dataset and model performance, including descriptive statistics, confusion-matrix results, and sources of misclassification such as code-switching and localized vocabulary. The section further interprets these findings in relation to prior research and established theories in sentiment analysis and SME digital transformation. It discusses the implications for model selection, language localization, and resource optimization, and elaborates on how the proposed **hybrid sentiment-analysis framework** enhances interpretability and adaptability—contributing both theoretically to text-analytics research and practically to SME decision-making in small-economy, multilingual environments.

**Section 7 – Conclusions** This section summarizes the major insights and contributions of the study. It outlines how the findings advance understanding of sentiment-analysis techniques within the SME context and suggests actionable recommendations for leveraging hybrid analytical models to improve customer insight generation and digital decision-making. The section also highlights study limitations and opportunities for future research.

**Section 8 – References** The final section lists all sources cited in the paper, formatted according to the journal's required reference style and encompassing both foundational works and the most recent (2020–2025) studies that inform this research.

### 1.2 Problem and challenges

As highlighted by Meske and Stieglitz (2013), social media has become an essential tool for small and medium-sized enterprises (SMEs), enhancing two-way communication with customers, improving internal knowledge sharing, and positively influencing employee morale. These benefits, along with the perceived ease of integrating social media into business operations (Bell and Loane, 2010), have encouraged many SMEs to adopt social media technologies to support their day-to-day activities. According to Pande et al. (2015) and Staudter et al. (2009), user-generated content such as posts and comments on platforms like Facebook is often considered the “voice of the customer,” as it reflects public sentiment and perceptions toward a business's products, services, and brand reputation. In Brunei, social media penetration is remarkably high—with over 516,500 active users reported in January 2022, exceeding 116% of the total population due to multiple accounts per user. Given such widespread usage, social media is no longer a supplementary channel but a crucial platform for business engagement in Brunei. However, while SMEs are increasingly leveraging social media for communication and marketing, many face significant challenges in extracting actionable insights from user feedback due to the sheer volume and unstructured nature of online content. Manual sentiment analysis becomes impractical due to time and resource constraints. Therefore, automated sentiment analysis using text mining offers a promising alternative.

Despite its potential, there is a clear gap in the existing literature regarding the practical application and effectiveness of automated sentiment analysis tools in the specific context of Brunei's SMEs. Most existing studies focus on broader or more developed markets, overlooking the unique linguistic, cultural, and operational challenges faced by SMEs in smaller nations like Brunei. For instance, social media comments often contain informal language, local slang, misspellings, and business-specific terminology that are not well-handled by generic sentiment analysis tools. These factors can significantly compromise the accuracy of automated methods, especially when dictionary-based models are applied without localization. Manual classification, while potentially more accurate in such contexts, is resource-intensive and not scalable. Therefore, there is a pressing need to investigate the limitations of existing sentiment analysis approaches and to identify whether a hybrid strategy—combining manual and automated methods—could provide more reliable insights for Bruneian SMEs. Addressing this gap is crucial for enabling SMEs in Brunei to better understand and respond to customer sentiment, enhance decision-making, and improve business performance in the digital age.

### 1.3 Research objectives

This study aims to evaluate and enhance the effectiveness of sentiment analysis techniques applied to social media content generated by Small and Medium Enterprises (SMEs) in Brunei. The primary focus is to assess the accuracy of dictionary-based text classification tools in analyzing customer comments on SMEs' social media posts, and to compare their performance with manual classification methods. This comparison is intended to provide insights into how effectively automated tools can identify trends such as customer demand, dissatisfaction, or general perceptions of the business. Additionally, the research explores the limitations of both automated and manual approaches by examining the unique features of customer feedback, including the use of slang, misspellings, off-topic remarks, and business-specific terminology that may influence classification accuracy. By understanding these limitations, the study seeks to determine whether automated sentiment analysis is a cost-effective and reliable solution for SMEs in Brunei, or if a hybrid approach combining both methods would be more beneficial within the local context. Therefore, the main objectives of this study are: (i) to evaluate the accuracy of dictionary-based sentiment analysis tools in classifying customer comments on social media posts by Bruneian SMEs compared to manual classification; (ii) to identify potential inaccuracies in manual sentiment classification of customer feedback; (iii) to analyze linguistic and contextual features in social media comments that affect the performance of automated tools; and (iv) to propose a feasible sentiment analysis framework that integrates both manual and automated methods to support SMEs in Brunei. Accordingly, this study aims to answer the following research questions: How accurate is the dictionary-based text classification approach for sentiment analysis of Bruneian SMEs' social media posts compared to manual classification? What are the potential inaccuracies in manual sentiment classification of customer comments? How feasible is it to combine both classification methods to support SMEs in understanding customer feedback? And how can classification methods be managed when dealing with informal comments—such as slang, misspellings, off-topic discussions, and business-specific language—that may affect the accuracy of sentiment analysis?

### 1.4 Significance of the study

By comparing the efficacy of manual and automatic text classifications in analyzing the sentiments or opinions expressed by SMEs in Brunei via social media, this study will add to the current understanding and application of sentiment analysis derived from these sources. This research has the potential to help small and medium-sized enterprises (SMEs) in Brunei by contributing to academic and literature of sentiment analysis purposes and its tools so they can better understand their customers' perceptions of their brand, company, goods, and services and allow more focus in developing and shaping the position of their business. Moreover, SMEs can gain insight into how their brands are regarded in the market by monitoring and evaluating social media sentiments and comparing them to their competitors. Improved services and product quality by SMEs, resulting in mutual benefits for both SMEs and customers, as well as higher GDP growth. This paper will focus on 5 different SMEs from different industries in Brunei. By utilizing their social media platforms and extracting comments and posts for text classifications to assess the accuracy of predicting sentiment categories they fall. This research employs a variety of software applications or platforms, including Facepager and browser extensions for data collection, RapidMiner Studio for tokenization, stop word reduction, stemming, normalization, and automatic classification, a dictionary with unique Facebook and Instagram post features, SentiWordNet 3.0 dictionary, and a Malay language dictionary. The goal of this research is to demonstrate how accurate text classification methods are for analyzing SMEs' social media platforms' sentiments, regardless of whether the classifications were made manually or automatically and to conduct an exploratory analysis of social media posts from SMEs in Brunei. That level of precision can be used to evaluate the viability of various business developments, including those aimed at expanding customer knowledge and service, and fostering creative problem solving.

## 2 Literature review

Sentiment analysis is a method that employs Natural Language Processing (NLP), statistics, and machine learning to extract and classify feedbacks from a textual input, often known as sentiment mining or the management of sentiment opinions and subjective text (Tul et al., 2017). These feedbacks can include recognition of subjectivity and polarity (Afful-Dadzie et al., 2014). A grasp of public sentiment can be gained through the use of sentiment analysis, which involves the examination of several tweets, reviews, and even comments on Instagram. It is an accurate strategy for predicting a wide range of significant events, such as the profitability of films at the blockbuster and the outcomes of general elections (Heredia et al., 2016). Automated sentiment analysis of particular texts is a subject of study that incorporates ideas from a wide range of fields (Kumar and Sebastian, 2012; Liu and Zhang, 2012). Several pieces of writing in the areas of retrieval of information, classification of texts, web mining and NLP have come about as a direct result of this. Subjectivity detection, sentiment categorization, and opinion summarization are only a few of the many subfields that make up the broader science known as “sentiment analysis.” Furthermore, since there is also a growing need to estimate and organize underlying data from social media in the form of unstructured data, there is a growing awareness of the necessity for sentiment analysis (El Rahman et al., 2019; Tul et al., 2017). Customers have continued to share textual material on social media that can be useful for sentiment analysis which will further improve the business practices and decision making (He et al., 2015). User-generated content creation and dissemination is facilitated by social media such as social networking platforms, virtual social and gaming worlds, collaborative projects, blogs, and others (Kaplan and Haenlein, 2010). Typical components of social media data include the user's username, the content that is published, the sequence of date or time of the post, the place that the user reports, connections to other individuals and online sources (Murphy et al., 2014). Initially, people utilized social media to stay in touch with friends and loved ones across great distances; this demographic was primarily comprised of students. As a result of this need, a plethora of social networking platforms have cropped up. Microblogging services, such as Twitter, gave users yet another method to keep their friends and followers updated on their day-to-day activities.

A significant role for social networking websites like Instagram, Twitter and Facebook has emerged in modern culture. On these websites, the users discuss anything and everything that is associated with their experiences, reviews, and opinions. In a similar vein, since businesses and organizations are likewise curious to learn customers' opinions regarding the quality of the products and services they provide, these entities could seek assistance from social media (Herhausen et al., 2019; Meire et al., 2019; Qamar et al., 2017). Classifying massive volumes of unstructured text data, such as posts on social media platforms, consumer reviews, and content published by companies, is an area that continues to draw a lot of attention because researchers want to learn how sentiment is related to important marketing outcomes (Netzer et al., 2019; Siebert et al., 2022) Text valence from social media has several applications; for instance, it can be used to predict stock market returns, impact consumer reactions to word-of-mouth, help predict customer lifetime value, and even be effective in social media (Meire et al., 2019; Tirunillai and Tellis, 2012). As mentioned by Abed et al. (2013) businesses are rapidly embracing and integrating social media technologies into their operations in order to assist in the production of value. To maintain competition, this is being done. Businesses are thought to be primarily motivated by external expectations from the external world, such as customers or competitors, to adopt social media (Parveen et al., 2015). Due to the development of social networks and external pressure, businesses use social media to easily communicate with their customers nowadays. Furthermore, small, and medium-sized businesses (SMEs) make extensive use of social media marketing since it has a low entry barrier in terms of both cost and technical expertise. According to Derham et al. (2011), In terms of their relationships with consumers on a personal level and the skills and expertise they have in the context of e-business, the owner-managers of SMEs are indeed a diverse group, and their use of social media networks is highly variable. For instance, SME could benefit from utilizing social networks like Facebook by reducing the expenses associated with delivering customer service, strengthening relationships with customers, and increasing access to information (Ainin et al., 2015).

In addition, the majority of customers and social media users will utilize social media to obtain product or service information or catalogues since it is resourceful, quick, and simple. Customers are known to publicly comment on social media platforms like Instagram, Facebook, and Twitter about their experiences with the goods and services provided by companies (El Rahman et al., 2019) and the wording used in posts on social media reveals several details. Specifically non-standard linguistic characteristics such as emoticons, online slang, various languages inside a single post, and spelling errors are common and should be considered (Laboreiro et al., 2010). This is due to the fact that customers are given the option of leaving reviews on specific products as well as general opinions on the platform because comments can be in multiple forms. Some companies do correspond with their social media posts, in which they frequently highlight particular products, services, or local events that they host, provide evidence of the strength of their relationship (Durkin et al., 2013) with customers that they have to easily attract potential customers who goes through their social media. Numerous factors affect the format and content of posts regarding small and medium-sized enterprises (SMEs) in Brunei. Small- and medium-sized businesses often only operate in a small geographic area. This usually leads to more open communication between businesses and their clients and staff. Additionally, posts on connected social media platforms that frequently reference certain products demonstrate a closer connection, services or community activities offered by businesses (Lee et al., 2008). According to a research by Jaman et al. (2020), shows an interesting investigation of how small and medium-sized businesses in Brunei are using social media. They believe that SMEs have quickly accepted social media as a chance to create two-way interactions with customers and create distinctive instruments for marketing communication. In addition, they said that social media has indeed been viewed as a vital player in Brunei's industrial development. In addition, social media is playing a significant part in the daily lives of Bruneians, since more than 86 percent of the population uses social media According to their research, SMEs in Brunei are comfortable whenever they are able to connect frequently with their clients via social media. In addition to increasing the number of clients who purchase their products or services, SMEs establish their reputation through social media. Businesses are able to predict the future purchasing behavior of their customers more accurately by modifying content that was created by customers themselves (user- generated content). As Kim and Ko (2012) pointed out, this enables companies to improve their brand recognition, which in turn helps them attract new customers, promote awareness, increase sales, and cultivate client loyalty. As user involvement grows and user-generated content becomes more prevalent, small businesses may not only benefit from a wide range of new options, but also find that many of the obstacles in their way are rendered moot. Using social media does not require a sizable financial investment and may be done at a low cost, as stated by Michaelidou et al. (2011).

### 2.1 Sentiment analysis

There are three distinct categories of sentiment analysis-based methods. The document- based algorithms attempt to classify the tone of a huge corpus of text, such as newspaper articles (Feldman, 2013). The second category investigates whether a independent word may be categorized as having a negative, positive, or having neutral polarity (Vohra and Teraiya, 2021). Aspect-based methods that concentrate on items and their properties are thought of as the third group (Liu, 2012). For instance, different products may have different qualities when they are reviewed. According to the research questions, the study's main goal is to investigate social media postings made by SMEs in Brunei in order to evaluate the precision of various text classification algorithms. Therefore, this research focuses on sentence-based methods, specifically dictionaries. When applying dictionaries to assess a text's sentiments, each item (word) is classified as either positive or negative (Medhat et al., 2014). There are a variety of ways that sentiment analysis may be carried out using social media, every one of which can provide organizations with a crystal clear picture of how critical it is for them to comprehend trends by evaluating sentiments (Pak and Paroubek, 2010) gave a thorough demonstration of how to use Twitter for sentiment analysis and opinion mining. Tweets written in English were rated on a three-point scale, from positive to negative to neutral, to determine their overall tone. All of the tweets were labeled by hand, with three different people doing the work. For the purpose of doing sentiment analysis (AbdelFattah et al., 2017) used the social media program Instagram. This type of analysis concentrates on analyzing the responses obtained by the top 50 clothing companies on Instagram given their top 20 pictures with the greatest number of likes. The goal of this type of analysis is to quantify the value of an image based on the input of comments, regardless of whether those comments are positive or negative.

Dictionary typically provide explanations of the meanings of the words they contain (Turney, 2002). When analyzing a paragraph, it is helpful to add up the scores awarded to each of its components so that you can get a sentiment of what the paragraph as a whole is trying to convey (Kundi et al., 2014). A corpus-based technique uses a domain-specific text corpus and different phrases to identify the sentence's context to determine the sentiment (Liu, 2012). Furthermore, to find recursively limiting negotiations or to ascertain the semantic direction of the sentences using adverbs and adjectives, depending on the sentiment analysis goal, are two ways this structure could be employed (Turney, 2002). Parallel operational units make up artificial neural networks, which classify sentence moods. Through weighted branches (Sebastiani, 2002), the phrases which need to be categorized move through the network. By changing branch weights, the networks can be taught. Sentiment analysis creates valuable information and data for businesses, especially SMEs because the nature of their business is small-to-medium therefore, they do not require IT specialist to collect data or perform this. This is because the software and programs can be easily built and found through the internet. It is also important for SMEs to have knowledge and understanding of the value of business intelligence for their business. Research by (D'Arconte 2018) brought attention to the practical use of business intelligence for small businesses. The profitability and customer happiness of every business were the authors‘ primary areas of concentration. They discussed clever technology that could aid businesses in enhancing their operations and increasing their chances of surviving and growing. In addition (Yue et al., 2019) study in this area has been distilled into three primary facets of the application, and it is mentioned that the increase in the amount of information that is available through social networking sites makes sentiment analysis increasingly important. When viewed from a business point of view, sentiment analysis could provide online guidance and suggestions for buyers and sellers alike. On the other hand, e-commerce platforms can use the customer preferences that are revealed by the data to assist them in conducting an analysis of their goods and services.

Customer relationship management, which should not be viewed solely as a technology in any way, is frequently linked to business intelligence. SMEs should be able to find intelligent technologies that may help them to survive a big market and competitors that are much bigger than them (Orji et al., 2022). In order to retain client relationships and loyalty, SMEs must be aware of and comprehend what customers want and want. One method to achieve this is to utilize the social media platforms that SMEs have for business analytics where sentiment analysis can be applied. Customer satisfaction is important when doing business because customers who are satisfied can be more loyal to the brand, product, or services the business provide. Businesses can learn more about their consumers and improve their services and products based on research gleaned from customers' opinions in a number of ways. He et al. (2015) has built a user-friendly social media marketing tool (VOZIQ) that gathers Twitter emotions to produce sentiments that can help organizations distinguish key performances, design promotional campaigns, and flag potential issue areas that need to be corrected in the organization. On the other hand, Jain et al. (2021) used sentiment analysis for online reviews of hospitality and tourism industries which can be used to design strategies for customers by prioritizing services that can fit demands of customers and therefore, reduce customer dissatisfaction.

### 2.2 Manual and automated classifications

Different types of classification tools have been utilized by various researchers. Some have utilized automatic categorization for sentiment analysis, while others may employ manual classification. The outcomes and objectives of many classification systems employed by researchers vary. They measured and evaluated the precision of their procedures in order to determine whether this strategy was successful or required additional development. Research by Al-Natour and Turetken (2020), they looked into whether automatic sentiment analysis might be used to determine the polarity of a product or service review. To do this, they assess the primary sentiment analysis methodologies' sentiment to accurately capture reviews' real feelings in addition to star ratings. They used contextual factors as two moderators influencing sentiment analysis accuracy. However, overall, sentiment analysis is quite good at determining the underlying meaning of the examined content and may be used as an alternative to or a replacement for star ratings. The results of their investigation of 900 reviews demonstrate that different methodologies that reflect the primary tactics of sentiment classification have differing degrees of accuracy. Wibowo et al. (2025) also investigated the use of sentiment analysis as a substitute for traditional ratings, which only give a numerical depiction of a show's success on television shows, was looked into. In order to analyze tweets for sentiment, they have used both automated and manual classification technologies like Lexicon-based and Support Vector Machine. The results show that these combined approaches can accurately analyze feelings up to 80% of the time. Their limitations noted that the two procedures combined had not produced satisfactory results. By adopting a vocabulary that can provide labels based on the degree to which each opinion term expresses positive or negative sentiments, lexicon-based approaches can be improved. For the Support Vector Machine approach, slack parameters must be used, or the dimensions must be changed to a higher dimension by utilizing another kernel, such as a polynomial or RBF. Greaves et al. (2013) applied a machine learning method to comprehend unstructured comments made by patients regarding their care. They classified patients' online free-text comments as either good or unfavorable descriptions of their health treatment using approaches for sentiment analysis. They had the option to forecast automatically if a patient would recommend a hospital or if the hospital was clean. They collect ~6,412 online comments regarding the hospitals and compare the results of sentiment analysis to those of a paper-based countrywide survey of hospital patients. The research evaluating the precision of manual and machine sentiment classifications was largely precise. Moreover, computerized classifications of surveys were more typical.

Gamon (2004) used training linear support vector machines to obtain high levels of automated classification accuracy in the difficult field of customer feedback data on software companies. Their approach heavily relies on machine learning, which has significantly increased the classification accuracy of feedback. The same data set (200 replies) was used for manual categorization as well, and identical outcomes (117 valid classifications) were discovered. The remaining material was either skimpy, nebulous, or unrelated to the main idea. The accuracy of automated categorization was 85%, whereas that of manual classification was 77.87%. According to their findings, using more abstract linguistic analytic characteristics regularly improves sentiment categorization accuracy. In order for the system to comprehend and deliver improved accuracy, more training and analysis of language samples are required. In order to assess a model that determines if an emotion is positive or negative based on comments made by Spanish peers, Ortega et al. (2020) conducted a study. A method of automated, supervised machine learning was developed to evaluate various combinations of N-grams using Term Frequency-Inverse Document Frequency (TF-IDF) and classification algorithms like Nave Bayes, Support Vector Machine, and others. According to the findings, the classification model using a support vector machine has the best accuracy, recollection, and F-measure values. Due to their limitations, it is advised to compare performance with other categorizing algorithms in order to find a model with promising outcomes.

The 2019 earthquake in Albania prompted (Contreras et al., 2022) to conduct research on a sentiment analysis classification model on tweets on emergency response and early recovery evaluation. Between November 2019 and February 2020, they collected 695 tweets using the hashtags #Albania, #AlbanianEarthquake, and #albanianearquake. They utilize the data to evaluate the precision of a sentiment analysis classification model that MonkeyLearn has previously trained to recognize polarity in text data. The test investigates whether it is possible to automate the classification process in the future to extract valuable information from text data from social media in real-time. Overall accuracy was 63%, with a 37% misclassification rate. The results of this experiment were regarded to be a preliminary assessment, and they will need to look for more chances to improve accuracy by changing the base classifier and machine learning system. Because they are employing hashtags to provide a more accurate and exact analysis, their restrictions indicated that there is a need to tailor their sentiment analysis for training to add an “unrelated” category. There are several researchers who have used automated classification tools for comments and reviews regarding SMEs through their social media. For small and medium-sized businesses and startups conducting market research and evaluating solely on the basis of customers' verbal feedback and demands, Orji et al. (2022) uses Machine Learning classification tools like Naïve Bayes, Linear Support Vector, and Logical Regression (LR) algorithm to determine the positive /negative text polarity of posts on Twitter. They demonstrated an increased performance accuracy of 83% for Logistic Regressions methods. The goal of this adoption was to gather customer viewpoints on their products or services.

Another example, is a research by Yennimar and Rizal (2019) they were able to categorize customer comments on their products from their website and social media and used a sentiment analysis approach with a machine learning classification algorithm to produce product rating value systems based on the overall view of a small and medium-sized enterprise (SME) in Indonesia The three classification methods used—Naive Bayes, Support Vector Machine, and K-Nearest Neighbor—gave results with an accuracy rate of 94%. Accuracy development is heavily dependent, nevertheless, on the limited information sources available for training and assessment. The objective of research into automatic text categorization is to develop models that classify new texts based on a training set of documents. For categorization purposes, documents are represented as document vectors, which are sets of features that characterize the document's content and style. The majority of prior studies on automatic sentiment classification emphasized “topical discussion” or “genre classification” classifications of texts (Pang and Lee, 2008) brought up machine learning techniques that have been successfully utilized for a long time to topical text categorization, they perform less well when used for sentiment classification. Comparing individual words in different subject areas to categorize articles according to the topic is a simpler undertaking than categorizing articles according to their sentiment. Several researchers prefer using manual classification tools for analyzing sentiment. Such as a research by Younis (2015) which have evaluated Twitter emotions of customers who posted online reviews of two of the largest retailers in the United Kingdom, namely Asda and Tesco. It was believed that the sentiment analysis of customer opinion would simplify the process for businesses to comprehend their market competitiveness in a competitive marketplace and to comprehend their customers‘ perspectives on their products and services, thereby providing insight into future marketing techniques and decision-making. They collected two thousand comments for Tesco and eight hundred and thirty tweets for Asda. They give score functions to tweets using a lexicon-based technique (manual classification). The results indicate that majority opinions are typically distributed when manually classified. However, they recommended that the unsupervised machine learning categorization techniques would be more beneficial. Wunderlich and Memmert (2020) provides a study evaluating the viability of lexicon- based sentiment analysis methods with respect to text information on the blogging network Twitter that is linked to football. Top ten football matches were mentioned in 10,000 tweets, which were submitted to both manual analysis by subject-matter specialists and machine sentiment analysis using readily accessible tools. The researchers' research demonstrates that a genuine collection of 1,000 tweets with a 60% share of tweets with the same polarity may be correctly categorized with much more than 95% reliability. Due to their limitations, it was inferred that applying suitable sentiment analysis techniques would result in classification accuracy that was more sophisticated and more accurate when classifying a single tweet or a small group of tweets.

In the research of Siebert et al. (2022), they find that the newly proposed transfer learning models perform the best but can perform significantly worse than widely used score board benchmarks using lexicon-based sentiment analysis, also known as dictionary-based sentiment analysis, in conjunction with 272 sets of data and 12 million sentiment-labeled text files. They show that transfer learning models identify documents more reliably by an average of 20% when compared to widely used lexicons. Schwaiger et al. (2017) conducted an intriguing study to develop a platform that allows SMEs in Germany to apply a language-appropriate automated classification approach for sentiment analysis. They arrived to the conclusion that due to the features of customer postings, such as the availability of language and accents dictionary-based techniques to posts are inadequate and machine learning is prone to make errors. Botchway et al. (2019) utilized a manual, Lexicon approach instrument for sentiment analysis. This instrument is tailored to the sentiments expressed on Twitter regarding UniCredit Bank of Europe. Using Valence Aware Dictionary and Sentiment Reasoner (VADER) to conduct sentiment analysis, it was determined that the overall discussion was positive, with a focus on tweets describing UniCredit's participation in socially responsible activities, promote for SMEs and entrepreneurship. According to Pang and Lee (2008) research on sentiment analysis using movie reviews, standard machine learning algorithms consistently surpass human-produced baselines. They have used three machine learning algorithms, though, including Naive Bayes, maximum entropy classification, and support vector machines, which they believe are less effective than conventional topic-based categorization at classifying sentiment.

### 2.3 Manual: Lexicon-based sentiment classification

Taboada et al. (2011) provided a system for classifying emotions using a lexicon as the basis for their research. It belongs to the category of unsupervised techniques for the classification of feelings. For the purpose of this classification task, they referred to word dictionaries that focused on either positive or negative polarity. By fusing intensifiers with negative terms, the Semantic Orientation Calculator (SO-CAL) was built using these dictionaries as a base. The lexicon-based method to movie review sets that (Liu and Zhang, 2012) developed revealed an accuracy range of 59.6% to 76% on a sample size of 1,900 papers. Developing sentiment lexicon may take some time and can be very difficult at the same time cannot be independently used to increase the viability of other techniques that is incorporated with them. There are three techniques to create a sentiment lexicon, however, this study will concentrate on methods that use dictionaries. By starting with a seed set of positive and negative words, doing WordNet expansion multiple times, and then training the classifiers on the enlarged set of positive and negative words (Esuli and Sebastiani, 2013), developed a method for categorizing words as positive or negative.

### 2.4 Automated: machine learning sentiment classification

Different approaches to conducting sentiment analysis via social media can be illustrated by using a variety of machine learning methods, which were developed through earlier research. The majority of the studies have applied the conventional categorization based on machine learning to the process of sentiment analysis. In this study, a variety of techniques will be used to evaluate the accuracy of machine learning techniques like Naive Bayes and Support Vector Machine (SVM). Both approaches have seen widespread use, particularly for the purpose of reviews, comments, and even feedback from individuals, with the goal of determining if the qualities associated with a sentiment are good or negative. According to Orji et al. (2022), Nave Bayes is a method that is based on Bayes'. Under the category of supervised machine learning, this kind of machine learning belongs to a classification technique based on the idea that the presence of one attribute has nothing to do with the presence of any other features. This model is straightforward to create and is of particular use when applied to very large datasets. In addition to its ease of use, the Naive Bayes method is widely acknowledged to be superior to even the most involved classification approaches. According to Ahmad et al. (2017) research, the accuracy of Naive Bayes when used for training and testing is higher than 80 percent. When it comes to the categorization of emotions, Orji et al. (2022) state that SVM is just as accurate as the Nave Bayes method. A performance accuracy rate of up to 80 percent can be achieved when assessing the sentiment of tweets with the use of remote supervision. Liu and Zhang (2012) see that the Support Vector Machine (SVM) has shown a little advantage over the Naive Bayes classifier in some scenarios. SVM is superior to generative models when it comes to the categorization of sentiments because of the superior ability of the former to differentiate between positive and negative emotions when both are present in the same comment.

### 2.5 Gap in literature review

Currently, the existing approaches and solutions lack the precision required for practical applications. This is especially true for posts on social media networks linked for Brunei context. Due to the presence of a large number of complex linguistic grammar rules in Brunei and Malay, a large number of techniques that were originally designed for English cannot produce acceptable levels of accuracy. Recognizing whether or whether a post contains sarcasm or regional language is particularly tough for the techniques. According to Kundi et al. (2014), many reactions to certain situations may excessively utilize slang and acronyms as a manner of expressing their sentiments. In addition, the results of their analysis propose the development of a system for identifying and evaluating Internet slang by combining SentiWordNet with other lexical resources. The thoughts may be perceived differently due to the prevalence of numerous internet jargons. More language analysis is required to establish a sample dictionary for categorizing distinct sentiments in SMEs posts. Understanding the specific issue that corresponds to the events associated with irony statements and being able to distinguish the emotions expressed by emoticons are essential for understanding irony. SMEs in Brunei currently do not have access to any cross-industry dictionaries with wide validity. This method is necessary for conducting sentiment analysis in practice. In conclusion, sentiment classification research is still in its mature stage, and marketable network monitoring technologies require additional improvement before they can be implemented to the advantage of SMEs in Brunei.

### 2.6 Research framework

**Figure 1:**
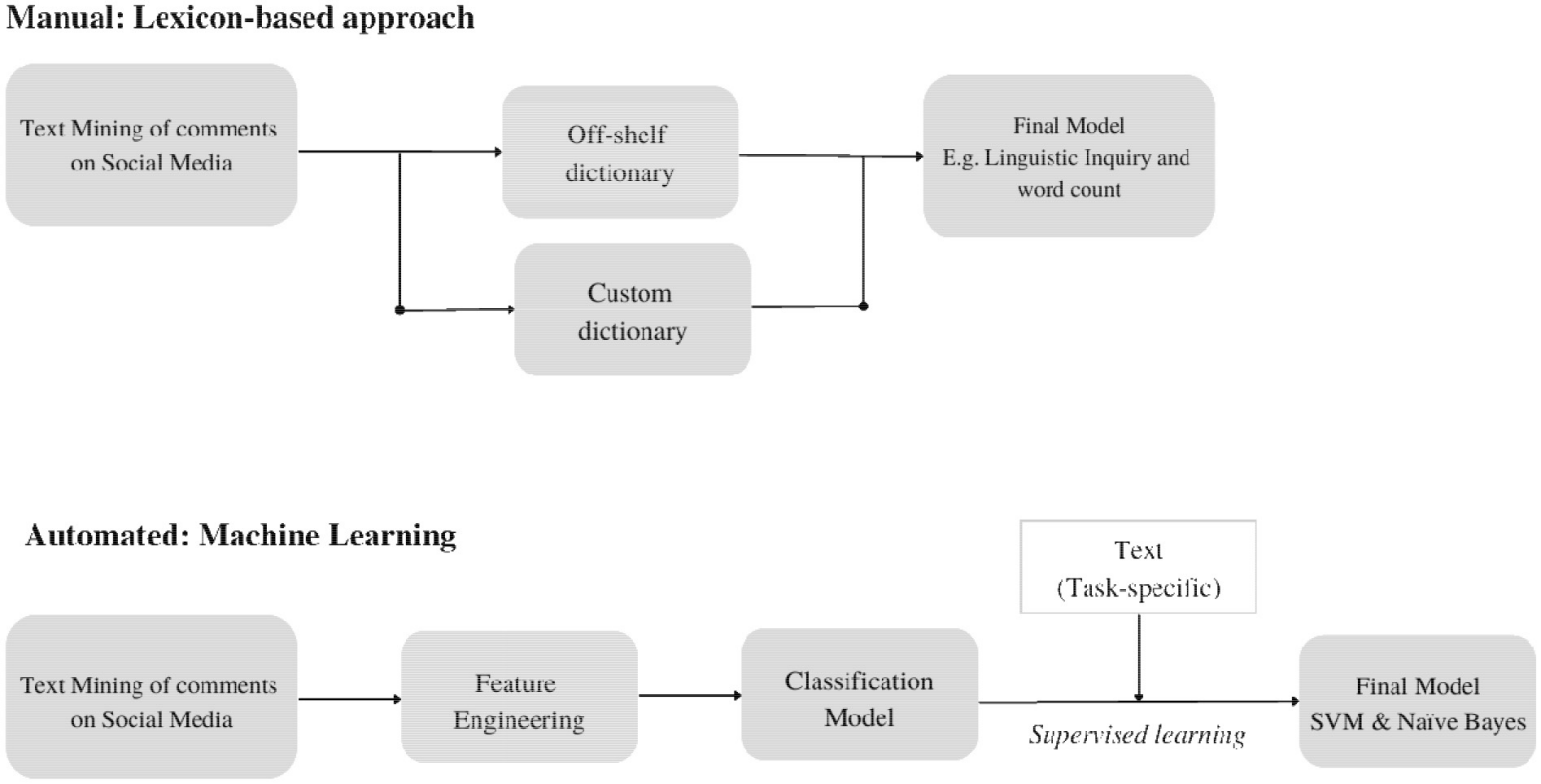
Research framework and guideline.

The three primary categories of methods are broken down in more detail in Figure 1. Following the example application of this research about sentiment analysis on social media for SMEs in Brunei, this summary will clarify the differences between the various approaches that were used to help in assessing their accuracy in the methodology stage. In applied research, sentiment lexicons are extremely useful and well-liked. A continuous emotion score or a sentiment orientation, such as positive and negative, is given to each string of words in a standard dictionary. Lexicons don't need labeled practice data. In its most basic forms, the program only counts the occurrences of words in the corpus and divides each document into classification based on the regularity of positive or negative words. Microsoft Excel will be used in this study's Lexicon-based approach. When less frequent or task-specific terminology is important, customized dictionaries can also be created in addition to off-the-shelf ones. Machine learning develops the ability to assign new observations with sentiments based on labeled training data. This approach is referred to as supervised learning since it involves learning from a task-specific label. Based on manually labeled data and a bottom-up analysis of the relationship between each word and the sentiment labeling of training data, machine learning techniques can automatically extract sample data word and sentiment connections. For each new application, the models are completely retrained. RapidMiner Studio will be used in this study along with a variety of operators, the Support Vector Machine (SVM), and Nave Bayes.

The selection of Support Vector Machine (SVM) and Naive Bayes classifiers in this study is based on their proven effectiveness and suitability for text classification tasks, particularly in sentiment analysis. Both algorithms have been widely used in natural language processing (NLP) due to their relatively simple implementation, strong theoretical foundations, and competitive performance across various domains. SVM is well-known for its ability to handle high-dimensional data and perform well in cases where the data has clear margins of separation, making it ideal for classifying polar sentiments such as positive or negative customer feedback. Its robustness to overfitting, especially when dealing with sparse feature spaces like bag-of-words or TF-IDF vectors, is a critical factor for sentiment analysis involving short, informal social media texts.

Naive Bayes, on the other hand, is a probabilistic classifier that performs efficiently even with relatively small datasets and under the assumption of feature independence. This characteristic is particularly useful for this research, where the goal is to classify a large volume of customer comments quickly and accurately, without the need for complex model tuning. Furthermore, Naive Bayes has been shown to yield competitive results in sentiment classification tasks, especially when dealing with unbalanced datasets—a common occurrence in user-generated content where neutral or positive sentiments often dominate.

Compared to more complex models such as deep learning approaches (e.g., LSTM or transformers), SVM and Naive Bayes require significantly less computational resources and training time, making them more feasible for implementation by SMEs in Brunei, who may not have access to high-performance computing infrastructure or specialized technical expertise. These algorithms also offer greater transparency and interpretability, allowing SMEs to better understand how classification decisions are made—an important aspect when justifying the use of sentiment analysis tools in real-world business decision-making. Therefore, SVM and Naive Bayes were selected for their balance of accuracy, efficiency, scalability, and practicality in the context of Bruneian SMEs dealing with local linguistic nuances and domain-specific terminology in social media content.

## 3 Research methodology

This study employs a mixed-method exploratory design with a strong emphasis on methodological rigor to evaluate sentiment analysis techniques applied to social media data from SMEs in Brunei. Data collection was carried out using automated web scraping tools—specifically Facepager and Instagram Comment Exporter—to extract publicly available posts and comments from the official Facebook and Instagram pages of five selected SMEs representing diverse industry sectors. This selection ensures domain relevance and diversity in business operations. The preprocessing pipeline, implemented in RapidMiner, includes several sequential steps designed to enhance data quality: tokenization to segment text into discrete units; normalization that involves lowercasing and removal of extraneous elements such as punctuation, URLs, emojis, and numerical digits; stop word removal to exclude common non-informative terms; and stemming based on the Porter algorithm to reduce words to their root forms. Additionally, a manually curated dictionary specifically developed to address Brunei-specific slang, misspellings, and bilingual Malay-English language variations is integrated into the preprocessing workflow. This dictionary is iteratively refined throughout the analysis to improve recognition of informal and localized language patterns, which are prevalent in user-generated content on social media platforms.

**Figure 2:**
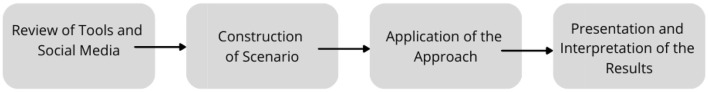
Research procedure.

For sentiment classification, the study applies two supervised machine learning models, Naive Bayes (NB) and Support Vector Machine (SVM), trained and tested on annotated datasets derived from the preprocessed social media texts. Hyperparameter tuning and cross-validation procedures are employed to optimize the models' performance. In parallel, a lexicon-based approach using a manually curated sentiment dictionary tailored to SME-related vocabulary and expressions common in Brunei's social media context is implemented for comparative analysis. Model evaluation relies on multiple performance metrics including precision, recall, F1-score, and accuracy to provide a comprehensive assessment of classification effectiveness. Furthermore, an error analysis is conducted using confusion matrices to identify common misclassification patterns, especially those caused by slang, sarcasm, and code-switching between Malay and English. This structured and replicable methodological approach allows for a detailed comparison between automated machine learning models and dictionary-based methods, offering empirical insights into the challenges and potentials of sentiment analysis in a bilingual, localized social media environment. The findings contribute valuable knowledge for future hypothesis-driven research and the development of more robust sentiment analysis frameworks tailored to SME digital communication (Figure 2).

The first step is the extraction of text or text mining from SME social media platforms, Instagram, and Facebook in this case. After the text has been extracted, it will undergo preliminary processing. Before entering the sentiment analysis phase, the corpus must undergo pre-processing. This is done to ensure that generalized words and phrases are used to measure the various sentiments and determine the most accurate method for classifying text. This indicates that the documents are prepared in a standard format with lowercase letters and no numbers. Text classifications can be categorized into 2 ways, manual and machine learning. This research will look into the different categories of classifications and make comparisons of how accurate it is to be used. Preparation of pre-labeled wordlist will also be used for manual classification and will then be used in RapidMiner for training and testing different datasets to ensure the accuracy of its performance. Machine learning will also be used to classify texts for sentiment analysis, and to see whether its accuracy levels are much different from those of manual text classifications.

The final outcome will then be evaluated based on its precision and the limitations of each categorization method. The stored data from each classification and analysis of sentiment will be utilized in the future as a reference to develop a deeper understanding of customers and influence business strategies. This study attempts to evaluate the precision of several manual and automated classification tools. In this part of the paper, we'll talk about how accuracy can be assessed. The number of items that were successfully detected overall in relation to the total amount of data being evaluated is a measure of the accuracy (Ayata, 2018). In other words, it represents the percentage of information that is accurate as opposed to unreliable throughout the process. In this instance, utilizing RapidMiner software's f- measure, precision and recall is to compare what is the anticipated true and the actual true of the sentiment classification process. RapidMiner will generate an output once the operations are complete to demonstrate the accuracy of the process and the system as a whole. This research will use descriptive statistics for data analysis. Data analysis will be done to study the dataset, find inconsistencies, spot underlying trends, make predictions, and come up with solutions that might make the process less accurate. To view the various sentiment polarities each categorization tool may analyze, the data analysis can be accomplished by employing statistical results with tables, figures, and graphic visualizations such as bar and line charts. Comparisons to the three categorization techniques can be conducted once data analysis has inputs (Table 1).

**Table 1:** Comparative summary table of reviewed literature.

Study/author(s)	Technique used	Domain/dataset	Key findings/ contributions	Limitations
Kumar and Sebastian (2012); Liu and Zhang (2012)	Sentiment Lexicon + NLP	General text datasets	Introduced foundations of sentiment analysis using polarity, subjectivity, and opinion mining	Primarily English; less effective for mixed-language texts
Pak and Paroubek (2010)	Manual labeling + Lexicon	Twitter (English)	Three-point sentiment classification (positive, neutral, negative)	Labor-intensive manual labeling
AbdelFattah et al. (2017)	Image comment sentiment analysis	Instagram (Top 50 clothing brands)	Quantified sentiment based on image post engagement	Ignores contextual nuances in visual content
Wibowo et al. (2025)	Lexicon + SVM	Tweets on TV shows	Combined methods reached ~80% accuracy	Combined approach still needs refinement
Gamon (2004)	SVM with Linear Classifier	Customer feedback on software	Automated classification achieved 85% accuracy	Some inputs were vague or off-topic
Ortega et al. (2020)	Naive Bayes, SVM, TF-IDF	Spanish peer comments	SVM showed highest accuracy, precision, and F-measure	Performance comparison with other models needed
Contreras et al. (2022)	MonkeyLearn-trained classifier	Tweets on 2019 Albanian earthquake	Achieved 63% overall accuracy; useful for real-time emergency sentiment tracking	High misclassification rate
Orji et al. (2022)	Naive Bayes, Logistic Regression, SVM	SME-related Twitter posts	Logistic Regression reached 83% accuracy	Limited to specific social media contexts
Yennimar and Rizal (2019)	NB, SVM, KNN	SME social media & website comments (Indonesia)	Achieved up to 94% classification accuracy	Dependent on limited training data
Pang and Lee (2008)	Naive Bayes, SVM, MaxEnt	Movie reviews	Machine learning outperforms human baselines	Genre/topic classification easier than sentiment classification
Taboada et al. (2011); Esuli and Sebastiani (2013)	Lexicon-based (SO-CAL)	Movie reviews	Developed semantic orientation scoring for dictionary-based classification	Lexicon creation is time-consuming
Younis (2015)	Manual Lexicon	Tesco & Asda Twitter comments	Lexicon-based manual scoring provided insight on customer preferences	Suggested moving to unsupervised ML methods
Wunderlich and Memmert (2020)	Lexicon vs Manual (Sports context)	10,000 tweets on football	Lexicon-based accuracy above 95% for large data volume	Less effective for small or ambiguous tweets
Schwaiger et al. (2017)	Lexicon & ML (Language-specific)	SME customer posts (Germany)	Found lexicon inadequate for dialects; machine learning prone to errors	Lack of language-specific resources

### 3.1 Data collection

The techniques and step-by-step methods for performing the data gathering will be provided in this chapter. The actions are a part of a bigger process that aims to create a social media monitoring tool that is specifically tailored to the requirements of SMEs in Brunei. There will be 5 different SMEs in different industries that will be used as a sample to extract their posts and comments. This will help control extraneous factors and sampling error by limiting the number of SMEs to use as sample. Instagram and Facebook will be the only social media network used in this paper to collect posts and comments for further sentiment analysis. Figure 1 Demonstrate the simple, step-by-step methodology of this study.

Reviewing various sentiment classification tools and social media that are relevant and appropriate for the process of extracting and crawling texts as well as sentiment analysis is the first step in identifying existing methodologies for sentiment analysis. This is because just a few techniques are appropriate for various social media posts, which represent a specific application area. As a result, the Instagram and Facebooks accounts of various SMEs in Brunei will be used as the social media platform for this research. The comments and posts collected through Instagram and Facebook because many businesses that are large or small-medium in size and users in Brunei use Instagram and Facebook as their main social media platform. Data can easily be extracted from the mentioned social media since majority are using that platform. This research article will use two distinct tools or applications for collecting data from the social media profiles of small and medium-sized businesses (SMEs) in Brunei. The first step is to use the IG Comment Export Google Chrome extension. This application only requires the links to the Instagram posts of SMEs in Brunei in order to retrieve all of the comments from such photos. It is utilized for the purpose of extracting comments and posts from Instagram. The second approach is to extract the Facebook posts and comments made by Bruneian SMEs through the use of a software program called Face Pager. The program only needs the Facebook user's login information as well as Facebook IDs and links in order to be able to extract the user's posts and comments from the social media platform. This article will also include references to the Tiny companies that can be found in other linked blogs that have developed more advanced methods for crawling and extracting data from Instagram and Facebook.

This study collected data from the official Facebook and Instagram pages of five selected Bruneian SMEs, all of which maintain an active and consistent presence on these platforms. These SMEs were chosen based on their public visibility, regular posting frequency, and engagement with followers. Publicly available business directories and social media activity were reviewed to ensure that only businesses with a sustained commitment to social media marketing were included. For each SME, approximately 200–300 user comments were extracted, resulting from a selection of around 100 recent posts (within a six-month period) deemed relevant to customer engagement and business performance.

To collect the data, we used **Facepager** for Facebook and **Instagram Comment Exporter** for Instagram. These tools enabled the efficient extraction of publicly accessible user comments for subsequent text mining and sentiment analysis. The focus was placed on posts that generated meaningful customer interaction—such as product inquiries, feedback, or service discussions—to ensure that the dataset accurately reflected customer sentiment.

The five SMEs selected for this study were carefully chosen to represent a cross-section of Brunei's small and medium-sized enterprise landscape, spanning various sectors with active digital engagement. Specifically, **Company A** operates as a food manufacturer and distributor, **Company B** is a leader in bottled water production, **Company C** serves a niche market focused on retailing low-waste and reusable products, **Company D** specializes in carpentry and bespoke woodwork, while **Company E** is an e-commerce platform advocating for sustainable and reusable living. These businesses were selected based on their industry relevance, active presence on social media (primarily Facebook and Instagram), and the availability of sufficient customer interactions in the form of comments and engagements.

The selected SMEs not only represent diverse industry domains—ranging from essential goods and manufacturing to eco-conscious retail and skilled trade services—but also reflect typical patterns of social media usage among Bruneian SMEs. Each of these businesses demonstrates consistent posting behavior and meaningful public engagement, making them suitable case studies for examining sentiment analysis applications. While the sample size is limited to five, this purposeful selection ensures contextual diversity and enhances the study's capacity to capture varied customer sentiment expressions and digital communication styles. As such, the findings can offer practical insights into the challenges and opportunities of sentiment analysis for SMEs across different sectors in Brunei.

However, several limitations and potential biases must be acknowledged. First, the data collection is restricted to publicly visible comments, excluding private messages or direct inbox communication, which may contain more nuanced feedback. Second, the analysis is limited to five SMEs, which may not fully represent the diversity of industries or digital behaviors across Brunei's entire SME landscape. Third, user-generated content often includes noise—such as off-topic comments, repeated emojis, or spam—that can affect sentiment accuracy and require careful preprocessing. Furthermore, the language used in Bruneian social media spaces often features local slang, code-switching between English and Malay, and informal spelling, which may reduce the effectiveness of generic sentiment analysis tools and require localized approaches or manual intervention. Despite these limitations, the collected dataset offers a practical and context-specific foundation for evaluating the performance and applicability of sentiment classification methods—both manual and automated—for SMEs operating in Brunei's digital economy.

## 4 Key finding

This research will be conducted on a total of five Bruneian SME businesses. Organizations have noted the need for automatic analysis mostly due to the massive volume of posts created by a significant number of followers. Table 2, displays the participating companies and the anticipated number of posts, mentions, and comments pertaining to them for the sentiment analysis study. All company names will be randomly changed to safeguard their privacy and maintain the confidentiality of research.

**Table 2:** SME Companies for this research.

Company	Description
Company A	Food Manufacturer & Distributor
Company B	Leaders of bottled water production
Company C	Niche market of retailing low waste & reusable items in Brunei
Company D	Leading in specializing carpentry
Company E	E-commerce platform to advocate reusable & sustainable living

### 4.1 Data sets

This research paper collects data from the Instagram and Facebook accounts of Brunei SMEs. All companies are registered businesses and are currently active in the business industry up to this day. This paper extracts texts from posts, comments, and Facebook statuses of SMEs. The total number of documents extracted is estimated to be between 2,000 and 3,000. The steps taken for the extraction and crawling of texts from SMEs social media are as follows:

- Search for Facebook and Instagram accounts from SMEs
- The extraction of text will have a duration of 2–3 years of posts, comments, and statuses from SMEs.
- Pre-processing of texts and preparation of pre-labeled texts are done through Microsoft Excel and Rapid Miner respectively.

Table 3 displays the number of Instagram and Facebook posts, status updates, and comments that were extracted. Each company's 100 most recent posts were extracted, including Facebook and Instagram status updates. The SME social media accounts on Facebook and Instagram yielded a total of 5,389 comments.

**Table 3:** Posts and comments extract from SMEs social media.

	No. of posts and comments extracted from Instagram and Facebook	
Company	Posts	Comments
A	100	1,540
B	100	536
C	100	689
D	100	1,492
E	100	1,132

Company A has the highest number of comments extracted from 100 posts, accumulating up to 1,540 (28.6%), while Company D has the second highest number of comments at 1,492 (27.6%). Furthermore, Company E has 1,132 (21%) and Company B and Company C have 536 (10%) and 689 (12.8%) respectively. However, the comments have not been pre-processed and filtered for the analysis of this research.

### 4.2 Exploratory stages

#### 4.2.1 Text mining

This paper will apply the idea using text mining techniques. Extraction of social media information from each company's Instagram and Facebook page is the first stage. This study will use an extraction tool based on browser extension and Face pager software to accomplish this goal. This tool connects to the current Instagram Facebook developer platforms and stores the gathered information in a common format. The texts and comments will go through a pre-processing and analysis stage after being extracted. Sentiment analysis algorithms must be modified to match the particular needs because social media posts have varied structural and linguistic properties.

#### 4.2.2 Pre-processing stage

This research will concentrate on the combination and recognition of emotes online terms, and many languages in a single statement, including Malay and English, utilizing RapidMiner Studio, which has operators that can pre-process and clean data. This is in addition to tailoring the algorithms for the briefness of social media posts. The procedure will then be able to undertake sentiment analysis on Brunei language social media posts that are specifically targeted towards the quirks of SMEs as a result. While Nave Bayes and Support Vector Machine will be utilized for automatic sentiment classification, a Lexicon-based technique will be employed for manual sentiment categorization. Data pre-processing is therefore necessary, and includes methods stemming, normalization, reducing stop words and tokenize. The process of breaking down posts into smaller units, such as single words, is known as tokenization. There are no longer any more symbols, punctuation, or special characters. The next step is stop word reduction. By doing so, phrases that do not express opinions can be removed from the discussion. These can be located using stop word lists that are available to the general public.

In this study, a comprehensive text preprocessing pipeline was implemented to ensure the quality and reliability of data before conducting sentiment classification. Using RapidMiner, several essential steps were followed: **(i) tokenization**, which splits the text into individual terms or words; **(ii) lowercasing**, to standardize all characters to lowercase for uniformity; **(iii) removal of stop words**, such as conjunctions and prepositions that do not carry meaningful sentiment; and **(iv) stemming**, which removes prefixes and suffixes to return words to their base or root form—e.g., reducing “standing” to “stand.” Additional cleaning involved the removal or normalization of **punctuation, emojis, URLs, and numeric digits** to minimize data noise.

Beyond standard preprocessing, this study addressed a major challenge in sentiment analysis of user-generated content: the high frequency of **slang, misspellings, and bilingual language mixing (Malay-English)**, especially in Brunei's social media context. To resolve this, we developed a **manually curated dictionary tailored to Brunei-Malay social media language**, including informal expressions, abbreviations, common typos, and industry-specific jargon. An **iterative update approach** was used—new variants discovered during the analysis were continuously added to the dictionary to enhance accuracy. This hybrid strategy allowed the model to adapt dynamically to the linguistic realities of Facebook and Instagram posts from SMEs in Brunei.

Furthermore, due to the informal and unstructured nature of social media comments, **feature extraction** was crucial. This involved defining key feature categories and selecting specific features relevant to sentiment interpretation. The use of domain-specific dictionaries ensured the inclusion of contextually significant terms that appeared frequently within the dataset, particularly those reflecting local expressions and business-related sentiments. This approach ensured the preprocessing phase not only cleaned the text but also preserved the nuanced information necessary for accurate sentiment classification in a Bruneian SME context.

#### 4.2.3 Classifications

A strategy that is going to be constructed in this article will be based on the dictionaries found in SentiWordNet 3.0. It is a lexical resource that may be used to automatically classify the feelings that people experience. SentiWordNet 3.0 is a lexical resource, although it is only available in the English language at this time. As part of this project, a Malay Language Dictionary will be compiled in order to make it easier for people to post and comment in Malay on social media platforms. The Malay Language Dictionary will be constructed by inserting pertinent Malay language words into the Malay perspective which are either synonyms or acronyms for English words that have been identified in the corpus. These words will be included in the dictionary. There will be two distinct text files, each of which will include both positive and negative Malay words in addition to a few English words. These words will be mixed in among the Malay. In order to increase the extent of coverage provided by the text file, Malay acronyms and synonyms that were discovered inside the corpus have been added to it. This was done.

#### 4.2.4 Evaluation

The data analysis and study findings are presented as the final phase in this process. The test data had to be manually evaluated before the outcomes of the automated application could be evaluated. The sentiment of each and every extracted post will be assessed for this reason in this research, and its value in comparison to automatic sentiment analysis will be evaluated in the assessment section. This paper will examine the manual feelings labeled as “reality” while attempting to minimize subjectivity. In this study, the reliability of sentiment evaluation will be measured in RapidMiner Studio using the well-known metrics of recall, f-measure as well as precision.

## 5 Data analysis and discussion

### 5.1 Results

After the following extraction of texts from Instagram and Facebook of the selected SMEs' social media, Table 4 depicts an overview of the comments and posts that have been pre-processed and filtered. Initially, there are irrelevant posts such as giveaways and product advertisement as well as posts of other languages other than Malay and English that were included in the extraction of posts on SMEs social media. Thus, the posts must be removed from the dataset, resulting in a reduction to exactly 60 Instagram and Facebook posts. There are a combined 300 from Instagram and Facebook across all companies with a total of 3,721 comments after the pre-processing stage.

**Table 4:** Overview of posts and comments.

	Number of posts and comments after pre- processing and reduction	
Company	Posts	Comments
A	60	1,120
B	60	286
C	60	349
D	60	1,134
E	60	832

The five companies were then subjected to the first text classification, which is Lexicon based sentiment analysis. The graph of the five companies demonstrates polarity scores of negative, neutral, and positive sentiments after including both Malay and English positive. For the purpose of determining whether or not the method is effective, this study will use three metrics that are quite well known: precision, recall, and f-measure. It is not possible to calculate the metrics without first defining the underlying variables. The approach divides the posts that are analyzed into one of three categories, based on their attitude: neutral, positive, or negative. This research needed to define a distinction between the two groups so that it could tell the difference between genuine and fictitious allocation of the relevant postings for each sentiment. As a consequence of this, we discovered six categories that do not agree with the conclusions drawn from the application of the automated methodology to real data shown in Table 5.

**Table 5:** Sentiment score categories and description.

Sentiment	Category	Descriptions
Positive	True Positive	Observations that are appropriately classified as having a positive attitude
	False positive	Observations that are classified as positive attitude but are not positive in real data
Neutral	True neutrals	Observations that are appropriately classified as having neutral attitude
	False neutrals	Observations that are classified with neutral attitude but are not neutral in the real data
Negative	True negative	Observations that are appropriately classified as having negative attitude
	False negative	Observations that are classified with negative attitude but are not negative in the real data

Table 6 displays the results of using dictionary-based approaches and the data that has been crawled and extracted from the five SMEs in Brunei. However, specific outcomes for various businesses and attitudes vary widely. Most of these distinctions may be traced back to the unique characteristics of SME social media posts, which are outlined in Table 7. Company A, the industry standard in food production and distribution, has, on average, f- measure values of 0.89, or 89%. The well-organized, precise, and exact language used in Company A's posts helps explain these high ratings. This is because the majority of the intended audience consists of business customers. This paper also provides examples of situations in which the adoption of the automatic technique generated findings that were merely neutral. Company E, an online shop that encourages recyclable and sustainable living, took into account the f-measure, which suggests an average of 69 percent hostile attitudes, 54 percent neutral feelings, and 55 percent positive sentiments. This is a significantly lesser amount when compared to all other companies. In the postings and comments pertaining to a certain topic, one can find a significant number of off-topic talks that have nothing to do with the products and services offered by the organization. During the initial round of manual classification, certain phrases that users employ when commenting on postings were considered to fall within the category of neutral sentiments. On the other hand, the automatic technique goes through each comment and reads it word for word without attempting to grasp the context of the statement. As a consequence of this, these posts are labelled as either good or negative based on the annotation of the words that are contained inside them. Another disadvantage is that consumers and members of the general public utilize lingo that is peculiar to the industry in their posts and comments on social media platforms, which the company has observed. Due to the fact that Company D is an industry pioneer in the field of carpentry, the positive set of datasets makes use of slang and jargon that are unique to the field; see Table 7 example 2 for an illustration of this. On the basis of these findings, it is required to improve the feature libraries that are currently available and add a number of recognized expressions of delight in addition to the watersports industry. When you understand the language that is used in the posts that are relevant to the industry, you will see that most of them express optimistic feelings.

**Table 6:** Results of Accuracy comparison between precision, recall and F-measure.

Organization participants		Real # of comments			Classified # of comments				Precision			Recall			F-measure		
		+	o	–	+	o	–	T	+	o	–	+	o	–	+	o	–
Company A	+	650			528	47	65	640	0.80			0.81			0.80		
	o		208		5	200	3	208		0.96			0.96			0.96	
	–				8	24	236	268									
				253							0.88			0.93			0.90
	T				541	271	304								Average: 0.89		
Company B	+	153			138	17	8	163	0.84			0.90			0.88		
	o		80		15	49	5	69		0.65			0.61			0.63	
	–				7	18	48	73									
				53							0.66			0.91			0.77
	T				160	84	61								Average: 0.76		
Company C	+	189			137	19	8	164	0.84			0.72			0.76		
	o		68		8	74	15	97		0.76			1.09			0.90	
	–				16	14	59	89									
				92							0.66			0.64			0.65
	T				161	107	82								Average: 0.77		
Company D	+	441			269	138	57	464	0.58			0.61			0.60		
	o		564	48	348	117	513		0.68			0.62			0.65		
	–			19	26	82	127										
			129							0.65			0.64				0.64
	T			336	512	256									Average: 0.63		
Company E	+	589			318	165	95	578	0.55			0.54			0.54		
	o		103		36	59	15	110		0.54			0.57			0.55	
	–				26	18	98	154									
				140							0.68			0.70			0.69
	T				380	242	208								Average: 0.6		

**Table 7:** Examples of limitations identified.

No.	Examples in Malay language	English language translation
1	v:♦ < 3	♦:v < 3
2	*Berapa harganya kalau yang cmani sis?*	How much are you offering for something like this?
3	*Baru ku cuba sekali servis membuat kayu dari dorang untuk my parents. Bisai eh, layanan pun terbaik…*	This is my first time trying their carpentry service for my parents. Well done, for the customer service as well..
4	*Bila kita kan buka kadai? inda semua orang pandai pakai online kalau kan shopping barang. Ada yang mau meliat dapan-dapan.*.	When are you going to open a physical shop? Not everyone can shop online. Some prefer to see the items physically before purchasing..
5	*Maskahih*	Spelling mistake (“makasih”)

The positive posts can be sorted into their appropriate categories with relative ease. Particularly companies whose material is published in a particular dialect that is indicative of the sector being discussed as a whole. For example, Company A and Company B both directly target the market with various goods and services, which resulted to high values for the f-measure of favorable sentiments within their respective customer bases. Additionally, as demonstrated in Table 7 example 1, the algorithm's accuracy improved as a direct result of the increased use of emoticons in social media posts as a means of conveying sentiments. Posts on social media regularly exhibit a variety of character traits by referring to many people. This may be seen, for instance, when users congratulate a company on opening a new branch in a different place or when they express a general opinion on particular events or awards (for an example of this, see Table 7 example 3). This occurred quite frequently for Company C, which excels beyond all others in its ability to specialize in carpentry. On the other hand, there are many different kinds of negative comments. The mean f- measure for positive posts is much higher than the aggregated f-measure for all posts, which was 60%, whereas the aggregated f-measure for negative posts was much lower. In contrast, the technique may have trouble recognizing negative posts due to the fact that these kinds of posts usually contain terms specific to a business and are written in an objective manner (refer to the Table 7 Example 4 for further clarification). Because of this, the value of the f-measure can end up being lower. On the other hand, this research indicated that a greater number of comments contained irony and negativity, which led to lower f-measure scores for negative statements. This finding contradicts the findings of a previous study.

In addition to that, it was found that the dataset had misspelt words in it. Because of the wide variety of people that use social media, misspellings are common in the posts that people make on these platforms. Due to the fact that the approach cannot identify incorrectly spelt expressions, the accuracy is greatly reduced as a result of this fact; see Table 7 Number 5. Simply said, “Thank you” is what “Makasih” translates to when spoken in Malay. In the Malay language, especially in Brunei, it is most commonly used as slang and a short form, and it is only rarely utilized as a general word.

### 5.2 Summary of data analysis

The F-measure results revealed performance variability across the five SMEs, with Company A achieving the highest score at 89%, and Company E the lowest at 60%. While dictionary-based models demonstrated strong accuracy when analyzing structured and grammatically correct language, their performance declined significantly in the presence of informal or inconsistent user-generated content. As shown in Table 6, accuracy dropped particularly when posts contained off-topic language, local slang, misspellings, or industry-specific terminology that were not captured adequately by the pre-trained sentiment models.

To better understand the limitations of the models used, an **error analysis** was conducted using **confusion matrices** to evaluate false positives and false negatives across classification categories. A key finding was the **high rate of false negatives**—especially in posts that featured **Brunei-Malay hybrid expressions**, informal abbreviations, or mixed-language commentary. These linguistic features often caused the model to misclassify negative or sarcastic sentiment as neutral or positive, due to limitations in both dictionary coverage and automated feature detection.

Moreover, **sarcasm and the use of emoticons** posed additional classification challenges. In several instances, sentiment cues were embedded in emojis or implied through sarcastic phrasing, which the model was unable to interpret accurately. The error analysis suggests that while automated techniques are efficient for large-scale sentiment evaluation, they may lack the nuance required to handle localized and expressive social media language without further training or contextual adaptation. This underlines the need for ongoing refinement of the sentiment analysis pipeline, particularly in datasets reflecting the linguistic diversity and digital behavior of Bruneian SMEs.

In summary, the sentiment analysis conducted in this study provides critical and actionable insights into the prevailing customer sentiments toward SMEs in Brunei. A predominance of positive social media posts can be interpreted as a strong signal of customer satisfaction and effective brand management, suggesting that the SMEs are successfully meeting market demands and fostering customer loyalty. Conversely, an abundance of negative posts typically signals underlying issues such as product dissatisfaction, service lapses, or unmet customer expectations, which require timely and targeted managerial interventions to prevent reputational damage. Thus, sentiment analysis emerges as an indispensable real-time barometer for SMEs to evaluate their operational success from the customers' perspective.

Table 5 highlights significant variability in sentiment classification accuracy across the five SMEs studied, with F-measure scores ranging from moderate to high levels, frequently outperforming SMEs' own expectations. This variability underscores the complex linguistic landscape present in Brunei's social media interactions—characterized by bilingualism (Malay-English), frequent use of localized slang, industry-specific jargon, and informal communication styles. These linguistic factors necessitate customized dictionary adaptation to ensure that sentiment analysis tools accurately capture the nuances in customer feedback, a task complicated by the hybrid language use prevalent among Bruneian social media users.

While automated sentiment analysis facilitates scalability and efficiency in handling vast volumes of customer comments, it also introduces unique cybersecurity vulnerabilities. Adversarial attacks—where malicious actors deliberately manipulate text inputs to confuse or deceive sentiment classifiers—pose significant risks. For instance, subtle text perturbations such as deliberate misspellings, insertion of contradictory slang, or use of sarcasm can lead to misclassification, potentially masking negative feedback or falsely inflating positive sentiment, thereby misleading business decision-making.

To address these challenges and enhance the robustness of sentiment analysis pipelines, this research integrates advanced adversarial defense strategies rooted in cybersecurity best practices. One such approach is **adversarial training**, which involves augmenting the training dataset with synthetically generated adversarial examples that mimic common text manipulations encountered in real-world social media posts. By exposing machine learning models to these perturbed inputs during training, the models learn to maintain accurate sentiment detection despite attempts to mislead them.

Additionally, **noise-injection techniques** are applied to artificially introduce controlled random perturbations during model training, which enhances the model's ability to generalize and resist unexpected input variations. Beyond these, the study proposes incorporating **text perturbation resistance mechanisms** such as character-level anomaly detection layers designed to flag unusual spelling patterns, repeated characters, or non-standard token usage indicative of adversarial manipulation.

Moreover, **semantic consistency checks**—which verify that the overall meaning of a comment remains logically coherent—can be embedded into AI pipelines to detect incongruities arising from sarcasm, irony, or mixed-language expressions common in Brunei's digital communication. These layered defenses are critical for SMEs, whose social media monitoring systems often operate with limited computational resources and are susceptible to subtle input distortions that could undermine sentiment analysis reliability.

Finally, by tailoring these cybersecurity-enhanced sentiment analysis frameworks specifically for the linguistic and cultural context of Brunei's SMEs, this research lays the groundwork for more resilient digital tools that support sustainable business growth, informed customer engagement, and proactive risk mitigation against adversarial threats in the evolving digital ecosystem.

To answer research question 3, from this research, The results of this experiment on the reliability of several categorization methods add to the existing body of knowledge in the field of sentiment analysis and are useful for small and medium-sized enterprises (SMEs) that use social media. The use of dictionaries as a starting point for research is well- established. There are a lot of dictionaries available online for automatic sentiment analysis, and they're all free to use. However, not everything is customized for certain companies or industries in Brunei. The Malay language also has limited lexical resources. In order to improve the precision of dictionary-based methods, vocabulary resources need to be converted from English to Malay and improved by considering the constraints of the companies that publish social media posts. In addition, the limits on the English language that have been obtained from study concentrate on the linguistic form of phrases that were initially designed to capture the slang of the language. By informing modifications to already existing dictionaries, these have the potential to make future iterations of Brunei's sentiment analysis more accurate. The number of people who use social media is continually growing, which broadens the range of applications that could be used for sentiment research. By doing so, existing dictionaries might be brought up to date, which would result in a significant increase in accuracy for sentiment analysis.

## 6 Conclusions

Automatic sentiment analysis was performed on the social media postings and comments made by five small and medium-sized businesses (SMEs) located in Brunei for the purpose of this research. This paper was able to extract, preprocess, and categorize posts from the business's Instagram and Facebook pages by using Face pager and extensions for Chrome. The outcomes show that a very high degree of accuracy was achieved. However, in order to obtain more accurate results, it is vital to customize the dictionaries to the language that is used both within a business and by the customers of that firm. Sentiment analysis, as was explained, allows for the automatic determination of the present attitudes of customers. This is especially helpful for small and medium-sized enterprises (SMEs), who often lack the manpower and time to keep track on their social media channels. Based on consumer sentiment data, a company can initiate initiatives to address concerns and improve their service.

The findings of this study can be useful for future research since they provide information on the impact that customer limits have on the social media posts made by SMEs. There is still a long way to go before we have a firm grip on how peculiarities of social media posts influence the accuracy of dictionary-based approaches. Due to its focus on SMEs in Brunei Darussalam, this study aided to fill a previously unfilled knowledge gap. According to the postings and comments, this resulted in the deficiencies of being brought to light. The outcomes of this study can be utilized by subsequent initiatives in order to better cater their methods to the individual requirements of SMEs. The personalization of dictionaries is one example of the things that fit under this category. Consequently, the findings will be utilized as input for the creation of algorithms that are capable of improved slang and irony recognition by, for instance, drawing on domain-specific expertise. The time commitment involved in manually extracting and crawling data as well as analyzing data for customer comments highlights the need for autonomous clustering solutions that are capable of completing this work in a timely manner.

## 7 Limitations and future directions

This study provides meaningful insights into sentiment trends associated with Bruneian SMEs through the application of both dictionary-based and machine learning-based sentiment classification techniques. Nevertheless, several limitations must be acknowledged. The analysis was based on data collected from five SMEs, which, although selected to reflect diversity in industry sectors and social media engagement, may not comprehensively represent the broader landscape of small and medium enterprises in Brunei. Additionally, data were drawn exclusively from Facebook and Instagram, thereby excluding other social platforms that may influence sentiment, such as TikTok or X (formerly Twitter).

Another limitation lies in the linguistic complexity of the dataset. Given the frequent use of Brunei-Malay and English in informal, hybrid forms, preprocessing and classification were challenged by slang, abbreviations, and code-switching. While efforts were made to mitigate this through a manually curated and iteratively updated dictionary, misclassifications—particularly in detecting sarcasm, implicit sentiments, and domain-specific jargon—remained evident in some instances.

Moreover, the generalizability of the sentiment classification models is constrained by the domain-specific characteristics of the training data. The performance of SVM and Naive Bayes classifiers, while effective within the study scope, may vary significantly when applied to SMEs in different cultural or linguistic contexts. Although adversarial defense strategies such as adversarial training and noise-injection were conceptually addressed, their practical implementation and evaluation were beyond the scope of this study.

Future research is recommended to expand the dataset to include a more diverse range of SMEs and incorporate additional social media platforms to better reflect evolving digital behavior. The development of real-time sentiment analysis dashboards integrated into business decision-making tools would enhance practical applicability. Further exploration into robust adversarial defense mechanisms, including text perturbation resistance and anomaly detection layers, may improve the resilience of sentiment models against manipulation or noise. Additionally, incorporating blockchain-based customer data frameworks may enhance data transparency, authenticity, and trust in sentiment insights. Finally, the application of advanced natural language processing models, such as transformer-based architectures trained on localized corpora, may offer improved handling of bilingual and culturally embedded expressions typical in Bruneian online communication.

## Data Availability

The original contributions presented in the study are included in the article/supplementary material, further inquiries can be directed to the corresponding author.
